# Cholangiocarcinoma: Consistent clinical, cytological, hematological, and biochemical findings and pathomorphology of the liver and kidney in five exotic dog breeds in Abeokuta, Nigeria

**DOI:** 10.14202/vetworld.2024.2053-2061

**Published:** 2024-09-15

**Authors:** F. M. Mshelbwala, O. L. Ajayi, A. A. Adebiyi, M. O. Olaniyi, T. M. Oladipo, E. F. Okpe, S. A. Rahman, A. F. Makinde, A. K. F. Kadiri, S. A. V. Abakpa, M. I. Olasoju

**Affiliations:** 1 Department of Veterinary Pathology, Federal University of Agriculture, Abeokuta, Nigeria; 2 Department of Veterinary Medicine, Federal University of Agriculture, Abeokuta, Nigeria; 3 Department of Veterinary Physiology and Biochemistry, Federal University of Agriculture, Abeokuta, Nigeria; 4 Veterinary Teaching Hospital, Federal University of Agriculture, Abeokuta, Nigeria; 5 Department of Veterinary Public Health and Preventive Medicine, Federal University of Agriculture, Abeokuta, Nigeria

**Keywords:** biochemical indices, cholangiocarcinoma, clinical signs, cytological findings, hematological findings, histopathology, malignancy, Nigeria, postmortem finding

## Abstract

**Background and Aim:**

Cholangiocarcinomas are malignant neoplasms that originate from any part of the bile duct epithelium. It is one of the most common liver tumors in dogs. This study described the clinical, cytological, hematological, biochemical, and pathomorphological findings of five cholangiocarcinoma cases in exotic breed dogs aged 2–5 years to aid in clinical diagnosis.

**Materials and Methods:**

This study used dogs presented at different times from 2012 to 2021 at the Veterinary Teaching Hospital, Federal University of Agriculture, Abeokuta. History, clinical signs, and vital parameters were recorded. Blood samples were collected for hematology and serum chemistry. Abdominocentesis was performed for cytological diagnosis. All dogs died during treatment, and postmortem examinations were performed. At postmortem, fine needle aspirates were collected from the liver and mesenteric lymph nodes and liver and kidney samples were fixed in 10% neutral-buffered formalin.

**Results:**

The dogs showed signs of severe malnutrition, jaundice, and bloating. The hematological analysis indicated anemia, neutrophilia without band neutrophils, and lymphopenia, indicative of a stress hemogram. The serum biochemistry test revealed lower levels of total proteins, albumin, and globulin and higher levels of serum enzymes. Abdominal fluid and mesenteric lymph node cytology revealed clusters of epithelial neoplastic cells. A postmortem examination revealed the liver’s nodular enlargement with the presence of button-like ulcers. Neoplastic epithelial cells are solid masses with hyperchromatic nuclei surrounded by fibrous connective tissues.

**Conclusion:**

Cholangiocarcinoma, diagnosed over a period of time in five exotic breeds of dog, consistently presents with the same clinical and postmortem findings, aiding in clinical diagnosis. However, the diagnosis of the disease is not possible in the early stage because of the absence of specific clinical signs. In dogs and possibly other animal species presenting with emaciation, lethargy, icterus, and distended abdomen, cholangiocarcinoma should be suspected, and cytological examination of the abdominal fluid and lymph node aspirates should be performed despite the absence of advanced equipment.

## Introduction

Cholangiocarcinomas are malignant tumors that originate from the bile duct epithelium [1–4]. The neoplasm exhibits both adenocarcinomatous structures (ductules and acini) and papillary formations. The cells display cuboidal or columnar shapes and possess a minimal quantity of slightly granular substance [1, 3, 4]. The disease affects dogs, cats, sheep, cattle, horses, and goats [5, 6]. They are classified as extrahepatic and intrahepatic; with the intrahepatic further divided as peripheral mass forming tumor and central periductal infiltrating tumor [1, 4, 7–13]. These three types of cholangiocarcinoma have been traditionally regarded as distinct disease entities clinically, therapeutically, and radiologically [1, 7]. Cholangiocarcinoma can show any of the typical exophytic, infiltrative, or polypoid growth patterns or combinations of the three patterns, and some can involve both the intrahepatic and extrahepatic ducts, which makes a clear classification difficult [4, 9, 12, 14]. Cholangiocarcinomas are all derived from the same biliary epithelium, exhibiting uniform biological behavior. Large bile duct tumors, due to their critical position, are detected early through symptoms such as jaundice or cholangitis. Small bile duct tumors do not obstruct bile flow until the advanced stage due to the tumor itself or metastases obstructing the common hepatic duct [9]. Cholangiocarcinoma can widely metastasize, thereby spreading to other organs such as regional lymph nodes, the diaphragm, intestines, pancreas, spleen, kidneys, urinary bladder, and the bone, giving a guarded to poor prognosis in affected animals [15, 16]. The clinical signs of cholangiocarcinoma in dogs include apathy, weight loss, inappetence, vomiting, increased urination and thirst, and swelling or massive distension of the abdomen, possibly due to a swollen liver or abdominal fluid [15, 17]. Gross pathological lesions include an enlarged liver with multiple nodular lesions and diffuse lymphadenopathy [17]. Histopathological lesions include an organized tubular or acinar arrangement in well-differentiated cholangiocarcinomas, and less differentiated cases show some acinar arrangements among solid masses of neoplastic cells. In contrast, poorly differentiated carcinomas comprise packets, islands, or cords of neoplastic epithelial cells [5, 12].

The present study is essential because the diagnosis of cholangiocarcinoma is difficult where there is no advanced equipment. Hence, there is a need to document the clinical presentation and postmortem findings in confirmed cases. The diagnosis of cholangiocarcinoma can be made based on the hemogram, serum biochemistry (hypoalbuminemia and elevated liver enzymes; alkaline phosphatase (ALP), gamma-glutamyl transferase, conjugated bilirubin and cholesterol, indicative of cholestasis), radiography, ultrasonography, computed tomography, magnetic resonance imaging, ultrasound-guided fine needle aspiration, and needle core biopsy. A clotting function test can also be performed [4, 12, 18]. However, a definitive diagnosis requires histopathological examination of biopsied tissue that differentiates it from primary hepatic carcinoma [18–21], which can occur concurrently in some dogs [12, 22, 23], and metastatic tumors from other organs. The treatment of cholangiocarcinoma can be a nonsurgical or surgical approach [16, 24, 25]. However, cholangiocarcinomas respond poorly to chemotherapy. Radiation therapy may be indicated postoperatively [26]. However, chemotherapy with doxorubicin or regional cryoablation can be used as palliative [27, 28] because these tumors have a guarded to poor prognosis and have frequently metastasized by the time of diagnosis [16, 29]. The study is essential to document the clinical presentation of cholangiocarcinoma observed in dogs in the present study, which can be used to help diagnose the disease, since clinical signs of cholangiocarcinoma can be confused with those in some other diseases. Early stages of the disease are reported to present with nonspecific clinical signs, while gross and histopathological lesions can present differently in cases of cholangiocarcinoma [5]. Also, it is worth mentioning that the disease can be diagnosed from fluid samples collected from lymph nodes, in addition to routine abdominal fluids.

This study details the clinical signs, laboratory findings, cytology, postmortem findings, and histopathology of cholangiocarcinoma in 5 exotic breeds of dogs, providing essential diagnostic insights when advanced diagnostic tools are unavailable.

## Materials and Methods

### Ethical approval

The study is a retrospective study. Data were retrieved from the record book of the Veterinary Teaching Hospital and the Department of Veterinary Pathology, Federal University of Agriculture, Abeokuta. The Veterinary Teaching Hospital is operated by trained and qualified Veterinarians who collected the blood samples using standard collection technique without harming the dogs. Therefore, Ethical approval was not applicable.

### Study period and location

The study was performed using data obtained from clinical records on five dogs with cholangiocarcinoma, presented for examination at different times, over a period of nine years (2012–2021). The dogs were presented to the Veterinary Teaching Hospital, Federal University of Agriculture, Abeokuta, Ogun State, Nigeria.

### History and clinical presentation

Five dogs with cholangiocarcinoma were diagnosed between 2012 and 2021 at FUNAAB’s Veterinary Teaching Hospital. Three were referred from private clinics, and two were presented directly. The patients’ histories were obtained, their clinical presentations were assessed, and relevant demographic information was documented.

### Physical examination and vital parameters of the dogs

On presentation to the Veterinary Teaching Hospital, physical examinations were performed, vital parameters such as temperature, heart rate, and pulse rate were taken, and body weights were recorded.

### Hematological analysis and abdominocentesis

In each case, blood samples were collected from the cephalic veins for hematological analysis using an automatic hem-analyzer (VH30, Genrui Biotech Inc., China), according to the manufacturer’s instructions. For cytology, abdominocentesis was performed in each case.

### Serum biochemical analysis

Biochemical analysis was performed using Randox test kits (Randox, UK). Total plasma proteins were determined using the direct Biuret method. Serum albumin concentrations were determined by the Bromocresol green method. Serum globulin was calculated as the difference between serum total proteins and serum albumin [30], whereas serum total bilirubin was determined according to the method described by Jendrassik-Grof method [31].

### Diagnosis and treatment

Diagnoses were made based on the history, clinical presentation, cytological examination of the abdominal fluid, and hematological and biochemical analyses. Palliative treatments were administered based on the confirmatory diagnosis. All the dogs died during the course of treatment and were presented for postmortem examination.

### Postmortem examination and histopathology

The postmortem examinations of the five dogs revealed gross lesions. Fine needle aspirates were collected from the liver and mesenteric lymph nodes for cytology examination. Liver and kidney samples from all cases were preserved in 10% buffered formalin for histopathological examination.

## Results

### History and clinical signs

The dogs comprised 2 Alsatian, 1 Rottweiler, 1 Bullmastiff, and 1 Cain Corso. They included 3 males and 2 females, aged between 2 and 5 years. The chief complaints in all the dogs were anorexia (4/5), weakness (5/5), lethargy (5/5), vomiting (3/5), icteric mucous membranes (5/5), emaciation (4/5), and distended abdomen (5/5) and melena. Before the presentation of the 3 dogs from the private clinics to the VTH, and cytological examination in all 5 dogs, they were being treated based on tentative diagnosis for babesiosis (45%), trypanosomosis (15%), ehrlichiosis (10%), parvoviral enteritis (5%), bacterial septicemia (15%), and worm infestation (10%) as the case may be; however, there were no positive outcomes.

### Vital parameters

The body temperatures ranged from 38°C–39°C; heart rates were between 110 and 145 bpm, while pulse rates were between 100 and 121 bpm. The dogs weighed between 22 and 32 kg (Table-1).

**Table-1:** Vital parameters in dogs presented with cholangiocarcinoma in the Veterinary Teaching Hospital, Abeokuta.

Vital parameters and breeds of dogs presented with cholangiocarcinoma						

Vital parameter	Alsatian	Rottweiler	Alsatian	Bull mastiff	Cain Corso	Reference value
Body temperature (°C)	39.0	38.4	38.0	39.7	38.8	38–39.5
Heart rate (bpm)	118	112	130	145	110	70–140
Pulse rate (bpm)	116	121	118	122	100	70–120
Body weight (kg)	26	28	30	32	22	Not available

### Hematological findings

The packed cell volume varied among the dogs from 18% to 28%. The total white blood cell (WBC) count varied slightly from 27.4 to 32.6 × 10^6^ μL, while the differential leukocyte counts also showed slight variation among the dogs, mainly neutrophilia and lymphopenia (Table-2).

**Table-2:** Hematological parameters and breeds of dogs with cholangiocarcinoma, presented to the Veterinary Teaching Hospital, FUNAAB.

Hematological parameters	Breeds of dog					

	Alsatian	Rottweilers	Alsatian	Bull mastiff	Cain Corso	Reference value
PCV (%)	21	27	18	28	25	37–55
Hb (g/dL) c	11.4	14.6	12.8	12.6	14.1	11.9–18.9
RBC (×10^6^/mcl)	3.88	4.57	3.48	4.96	4.89	4.95–7.87
Plasma protein (g/dL)	3.56	4.38	3.28	4.88	4.12	6.54
Plasma color/transparency	Clear	Clear	clear	Clear	Clear	Clear
Total WBC (×10^3^/μL)	28.9	29.1	32.6	26.8	27.4	5.0–14.1
Neutrophil (Absolute)	8,942,132	8,652,340	8,251,301	7,983,451	8,876,600	8,354,341
Band neutrophil (Absolute)	0.00	0.00	0.00	0.00	0.00	0.00
Lymphocytes (Absolute)	1,287,453	1,148630	1,324,510	1,421,352	1,507,000	6,313,243
Basophils (Absolute)	0.00	0.00	0.00	0.00	0.00	0.782674
Monocytes (Absolute)	412	311	234	302	0.00	0.182987
Eosinophil (Absolute)	0.00	0.00	0.00	0.00	0.00	0.235623
MCV (fl)	67.4	69.8	64.4	68.6	68.4	66 –77
MCHC (g/dL)	34.6	33.2	34.6	35.1	34.1	32.0–36.3
MCH (pg)	24.6	22.3	21.5	23.7	22.6	21.0–26.2

PCV=Packed cell volume, Hb=hemoglobin, RBC=Red blood cell, WBC=White blood cell, MCV=Mean corpuscular volume, MCHC=Mean corpuscular hemoglobin concentration, MCH=Mean corpuscular hemoglobin

### Serum biochemical findings

In all five dogs examined, serum biochemistry showed decreased glucose, total protein, albumin, and globulin concentrations. Meanwhile, conjugated and unconjugated bilirubin increased markedly. Furthermore, serum enzyme concentrations increased, including serum glutamate oxaloacetate transaminase, serum glutamate pyruvate transaminase (SGPT), aspartate aminotransferase (AST), ALP, and alanine aminotransferase (ALT) (Table-3).

**Table-3:** Biochemical changes associated with cholangiocarcinoma in dogs in Abeokuta, Nigeria.

Parameters	Alsatian	Rottweiler	Alsatian	Bull mastiff	Cane Corso	Reference	Inference
Carbohydrate (mg/mL)							
Glucose	13	27	18	34	23	75-120	HG (5/5)
Proteins (g/L)							
Total protein	67.8	58.6	48.8	54.7	63.1	60-80	HP (3/5)
Albumin	34.1	31.4	28.3	32.6	36.7	40-60	HA (5/5)
Globulin	33.7	27.2	20.5	22.1	26.4	20-30	HGl (4/5)
Bilirubin (mg/dL)							
Total bilirubin	34.1	36.5	28.4	31.9	33.8	0.5-1.5	HB (5/5)
Conjugated	27.0	25.3	18.6	21.7	25.0	0.1-0.5	HB (5/5)
Unconjugated	7.1	11.2	9.8	10.2	8.8	0.1-0.5	HB (5/5)
Enzymes IU/L							
SGOT	45.7	57.3	48.6	63.5	58.4	0-21	⇧ SGOT (5/5)
SGPT	14.8	43.2	24.5	38.8	28.4	0-18	⇧ SGPT (4/5)
AST	348.3	397.8	456.4	398.5	478.6	10-60	⇧ AST (4/5)
ALP	217.5	382.3	289.5	302.4	268.1	60-160	⇧ ALP (5/5)
ALT	357.4	478.8	356.3	389.5	298.4	10-125	⇧ ALT (5/5)

⇧=Increase, SGOT=Serum glutamate oxaloacetate transaminase, SGPT=Serum glutamate pyruvate transaminase, AST=Aspartate aminotransferase, ALP=Alkaline phosphatase, ALT=Alanine aminotransferase, HG=Hypoglycemia, HP=Hypoproteinemia, HA=Hypoalbuminemia, HGl=Hyperglobulinemia

### Cytological findings

**Figure-1:**
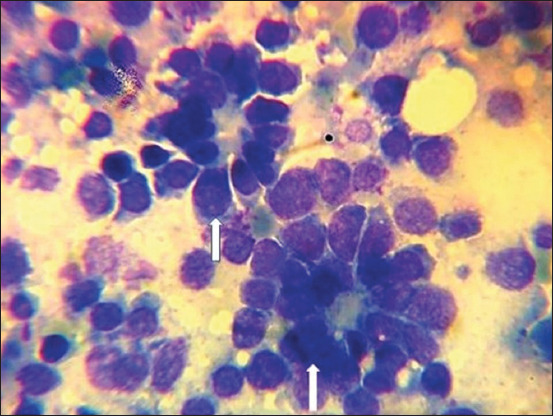
Cytology of abdominal fluid revealed neoplastic cells of epithelial origin appearing in clusters, forming acini and showing pleomorphism, hyperchromatic nuclei with high nuclear-to-cytoplasmic ratio (arrows) (1000× Giemsa stain).

The cytology results revealed neoplastic cells of epithelial origin appearing in clusters and forming acini in all five affected dogs (Figure-1).

### Preliminary treatment

In each case, preliminary treatment was instituted with Dexamethasone (JAT, International DMCC, Dubai), 1 mL, injection, intramuscular, once in a day, for one week; Augmentin (Medreich Limited, India), 5 Ml, injection, intravenous, twice a day, for 5 days; Vitamin K (Guizhou Tiandi Pharmaceutical company Limited, China), 2 mL injection, intramuscular, once in a day for 5 days; in two of the dogs. While Ciprofloxacin (R.K. Laboratories (P) Limited, India), 10 mL intravenous twice a day, for 5 days; Vitamin K (Guizhou Tiandi Pharmaceutical Company Limited, China), 2 mL injection, intramuscular, once in a day for 5 days and Isoplasma (Unique Pharmaceuticals Limited, Nigeria), were used in the other three dogs. However, all the dogs died during treatment.

### Postmortem findings

**Figure-2:**
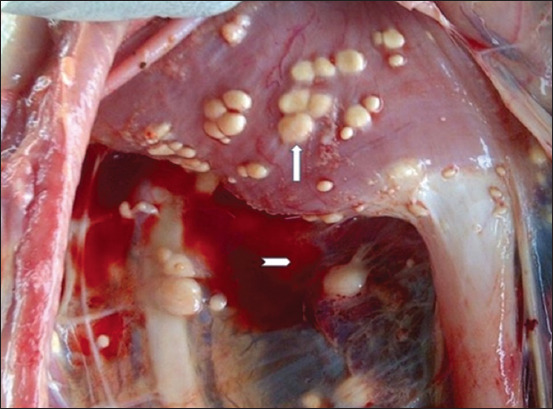
Presence of whitish to tan nodules in the diaphragm (arrow) and hydroperitonium (arrowhead).

**Figure-3:**
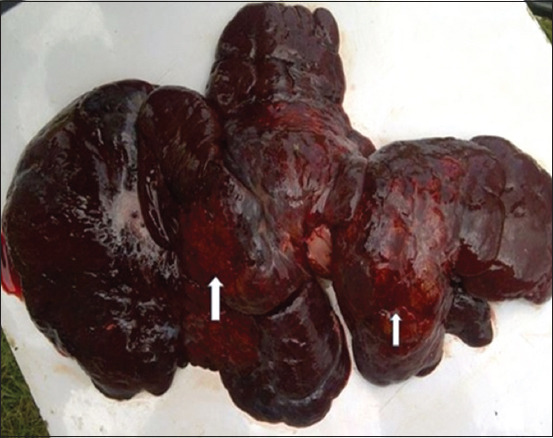
Markedly enlarged liver, 5.4 kg, showing nodular and necro-hemorrhagic surfaces (arrow).

**Figure-4:**
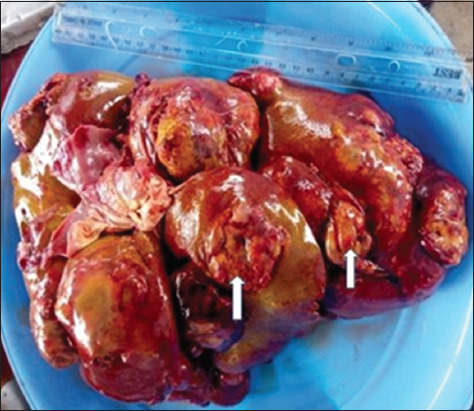
Markedly enlarged liver (3.5 kg) with numerous varying sizes, discrete to coalescing, rough-surfaced ulcerated (button-like) nodules (0.2 cm × 0.2 cm–6 cm × 6.7 cm) projecting from the surface (arrows).

The dogs were presented for postmortem examination within 2–4 h after death. The carcasses were emaciated (4/5) and had distended abdomens (5/5); the ocular and oral mucous membranes were markedly pale but masked by icterus (5/5); and the subcutaneous icterus was observed (5/5). The regional lymph nodes were markedly enlarged and nodular (5/5), the diaphragm had yellowish to whitish multifocal nodules (3/5) (Figure-2), and the thoracic and abdominal cavities contained a large volume of serosanguinous fluid, measuring between 1 and 3 L (5/5). The lungs were severely congested and edematous (5/5). The livers were markedly enlarged, nodular, and ulcerated (5/5) (Figures-3 and 4). The spleens were diffusely enlarged, saggy, and pulpy (5/5). The kidneys were enlarged and nodular (3/5). The stomach contained scanty fluid and the entire intestine contained scanty feed and fecal materials (3/5).

### Histopathological findings

**Figure-5:**
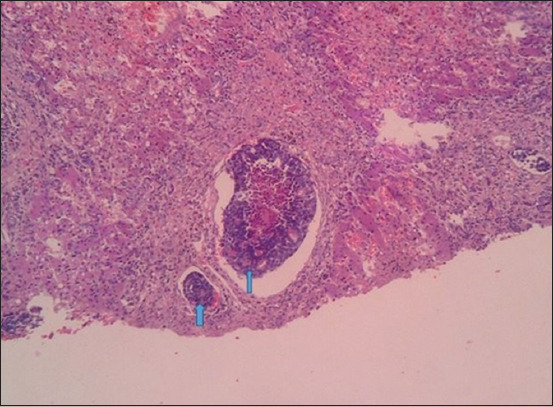
Section of the liver showing neoplastic cells that are stained deeply basophilic (hyperchromatic) and solid masses of neoplastic cells were separated by fibrous connective tissues (arrows) (10× Hematoxylin and eosin).

**Figure-6:**
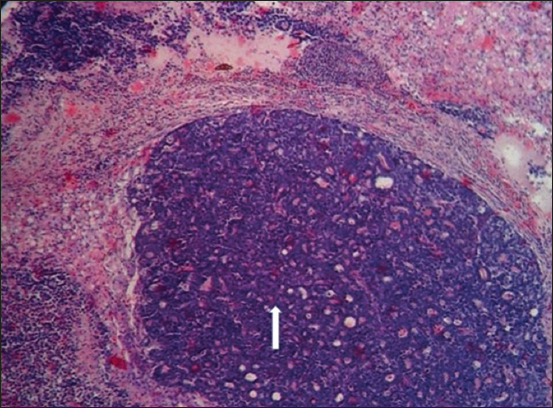
Section of the liver showing neoplastic cells that are stained deeply basophilic (hyperchromatic) and solid masses of neoplastic cells were separated by fibrous connective tissues (arrow) (400× Hematoxylin and eosin).

**Figure-7:**
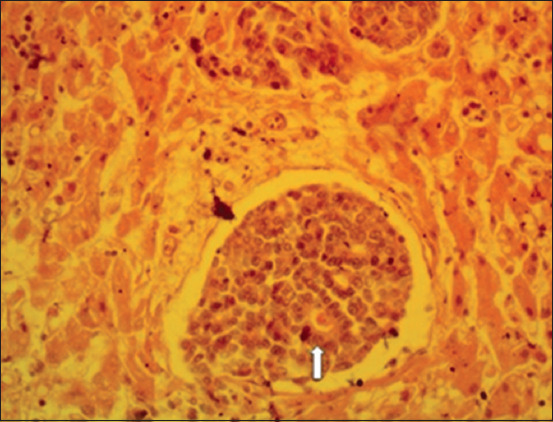
Section of the liver revealed acinar arrangement of tumor cells among solid masses of neoplastic cells, with mucin within their lumen (arrow) and vacuolar degeneration of compressed hepatocytes (arrow) (40× Hematoxylin and eosin).

**Figure-8:**
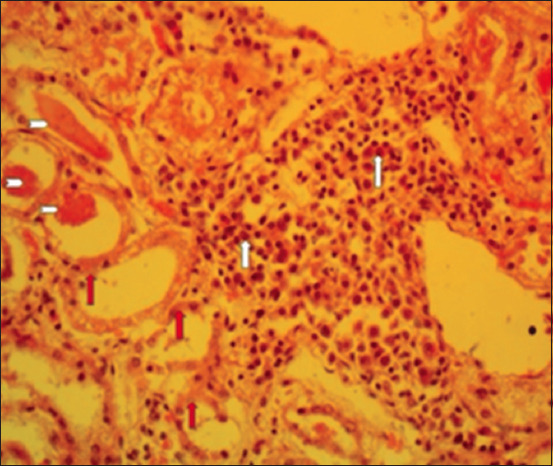
Section of the kidney showing a focal interstitial mass of pleomorphic cells (white arrows), the necrotic tubular epithelium (red arrows), and intratubular eosinophilic cast (arrowheads) (400× Hematoxylin and eosin).

Section of the liver showed neoplastic cells that were stained deeply basophilic (hyperchromatic), and solid masses of neoplastic cells were separated by fibrous connective tissues (Figures-5 and 6). In other cases, the liver section showed an acinar arrangement of tumor cells, with mucin within their lumen and also separated by fibrous connective tissues. The tumor masses compressed the surrounding hepatic cells, resulting in vacuolar degeneration (Figure-7). Section of the kidney showed focal interstitial masses of neoplastic cells that appeared pleomorphic. The tubular epithelium was necrotic and there was an intratubular eosinophilic cast (Figure-8).

## Discussion

Cholangiocarcinoma, which is a life-threatening disease [32], is the second most common type of liver tumor that affects dogs. The population characteristics of the affected dogs in this study showed that different breeds, ages, and sexes are susceptible to the disease. Breeds have been reported to predispose dogs to cholangiocarcinoma, as observed in Labrador retrievers, although other causative factors are implied in the occurrence of cholangiocarcinoma; however, there was no breed predisposition to cholangiocarcinoma [33]. It has also been reported that human biliary tract cancer risk is not associated with a familial predisposition and may be mitigated more strongly by environmental modifiers [34]. On the other hand, cholangiocarcinoma has been found to be more common in female dogs and in dogs that are 10 years of age or older [33]. In this study, different breeds, both sexes, and young and older dogs were affected.

The cause of the disease in this study is not known. It has been reported that the etiology of bile duct tumors remains poorly understood. However, it has been associated with *Clonorchis sinensis*, a trematode frequently found in natural and experimental cases in cats and humans in Southeast Asia [35] and with hookworms and/or whipworms [36]. In addition, prolonged exposure to aminoazotoluene and aramite, a sulfur-containing compound used for insecticide purposes, may cause cholangiocarcinoma, as reported by Cullen and Poop [5]. Other risk factors for bile duct tumors in humans are bile duct stones and sclerosing cholangitis [35, 37]. Hepatitis B and C viruses, which cause chronic hepatitis in humans and laboratory animals; Woodchuck hepatitis virus; and mycotoxins, such as anatoxins, have also been shown to cause hepatobiliary tumors [17, 38, 39].

The clinical signs such as icterus, cholestatic syndrome, abdominal distention, ascites, anorexia, weight loss, lethargy, and vomiting observed in the present study are consistent in all 5 cases and also with those reported in the literature [15, 35, 37, 40–42]. Inappetance, vomiting, and weight loss have been reported in a Siamese cat with cholangiocarcinoma of the intrahepatic bile ducts that showed disseminated metastases [43]. However, most of the clinical signs can be confused with those observed in hemoparasitic infections and endoparasite infestations. Cullen and Poop [5] reported that early diagnosis of cholangiocarcinoma is not possible because of the absence of specific clinical signs. In humans, symptoms of biliary tract cancer include icteric syndrome and right hypochondrium pain (cholestatic syndrome) [42].

Vital parameters recorded in all 5 cases in this study were similar and within the normal ranges. Neoplastic conditions may present with normal body temperature, pulse, and heart rates [44].

Hematological parameters associated with cholangiocarcinoma in this study revealed consistent findings, the hallmark of which was anemia. It has been reported that dogs with cholangiocarcinoma usually suffer from anemia and leukocytosis, a condition characterized by an elevated number of WBCs and thrombocytosis, which is an abnormally high number of platelets in the blood [41]. In the present study, except for thrombocytosis in which platelet count was not performed, there was moderate-to-severe anemia, leukocytosis, characterized by neutrophilia without left shift (band neutrophils), monocytosis, and lymphopenia, which suggest a stress hemogram due to the effect of the neoplasm, and possible inflammatory reaction.

Serum biochemistry profile, AST, ALT, ALP, and total bilirubin (conjugated and unconjugated) were high, which was similar to a previous report by Thamm [35] in dogs with cholangiocarcinoma. Elevated serum ALP is generally considered as an indicator of hepatobiliary disease, such as choledocholithiasis [45], bile duct strictures, and malignancy [46]. AST, ALP, glutamic oxaloacetate transaminase, ALT, and SGPT are useful indicators of hepatocellular injury and/or muscle injury in large and small animals [46]. In the present cases, there was no evidence of muscle damage. Changes in the serum biochemistry profile of dogs with hepatic tumors may also indicate hypoglycemia and hypoalbuminemia, as seen in the present cases. An increase in total bilirubin can be caused by hemolysis or cholestasis; more so, anemia was evident and the type of anemia in this case could either be hemorrhagic or hemolytic anemia. Evidence of hemorrhagic anemia includes hemoperitoneum and melena.

On the other hand, the clear appearance of plasma precluded the possibility of hemolysis. Therefore, the increase in total bilirubin was probably due to hepatobiliary disease and secondary to blockage of bile flow (cholestasis), as the tumor masses compressed or blocked the bile ducts [42]. Bilirubin is considered a test of hepatic function; in essence, the ability of the hepatocyte to take up unconjugated bilirubin in blood, conjugate it (render it water soluble), and excrete bilirubin into bile, where it is broken down in the intestine by bacteria. However, in reality, bilirubin is not used as a test of the functional capacity of the liver (rather bile acids and ammonia are the more common tests used for this) but more as a marker of liver disease (with and without cholestasis); that is, to support a diagnosis of hemolytic anemia. Bilirubin is an antioxidant, its main physiological function [47]. However, symptoms of biliary tract cancer in humans have been reported to include icteric syndrome and right hypochondrium pain, which is a cholestatic syndrome [42].

Cholangiocarcinoma can metastasize to regional lymph nodes, diaphragm, intestines, pancreas, spleen, kidneys, omentum, urinary bladder, and bone among other organs [15, 16]. In these particular cases, metastasis to the omentum, pancreas, diaphragm, and mesenteric and hepatic lymph nodes were observed. Gross lesions of the hydroperitoneum, nodular enlargement of the liver with button-like ulcers, and jaundice observed in the present cases were consistent and similar in all five cases. Enlargement of the liver is a consistent finding in cholangiocarcinoma [39]. The general necropsy findings in cholangiocarcinoma are usually one or more firm and white masses that are slightly deflated in the middle, papillary, and tend to be well-delineated round or cauliflower-shaped masses located on the liver surface. The sectioned surface of the neoplastic tissue is white and may include necrotic and hemorrhagic areas [48].

Histopathological findings showing the acinar arrangement of tumor cells, with mucin within their lumen and vacuolar degeneration of compressed hepatocytes, were consistent in all five cases. Nakanuma *et al*. [4] reported that cholangiocarcinoma presents an exophytic growth in the dilated bile duct lumen and shows villous (papillary) neoplastic epithelia, with tubular components covering fine fibromuscular stalks. All of the present cases appeared to be intrahepatic cholangiocarcinomas, which are characterized by tubular structures lined by anaplastic cuboidal or columnar cells with diffused fibrous stroma. Bile duct cyst adenocarcinoma is characterized by multiple cystic structures with papillary and solid masses.

Diagnosis of cholangiocarcinoma is difficult in live animals in developing countries because of the unavailability of sophisticated equipment. Tissue collection during endoscopy and/or percutaneous transhepatic procedures is usually used to confirm the diagnosis of cholangiocarcinoma, but the procedure is tissue invasive, risky, and requires considerable technical know-how. In addition, forceps biopsy and brush cytology can provide positive results for malignancy in only about 50% of patients [49]. Moreover, although increased serum concentration of liver enzyme is an indicator of hepatocellular injury, this cannot be used in the diagnosis of cholangiocarcinoma because there are many causes of hepatocellular injury and corticosteroids which cause exogenous or endogenous “stress” can induce an increase in this enzyme. However, there was no history of corticosteroid administration in any of the five cases examined. On the other hand, cytological evaluation, postmortem examination, and histopathology are easy to perform and are used to diagnose cholangiocarcinoma [48]. In this study, cytological examination of the abdominal fluid collected through abdominocentesis demonstrated neoplastic cells of epithelial origin, suggesting that they were cholangiocarcinoma cases.

Treatment of cholangiocarcinoma in the present cases was palliative, targeted at resolving peritonitis, dehydration, and hemorrhage. Management strategies include multispecialty treatments, including surgical resection, systemic chemotherapy, and radiation therapy [10, 25]. Surgical removal of localized intrahepatic bile duct carcinoma in dogs that showed a good prognosis has been reported [16, 21]. Adjuvant therapy with toceranib for hepatocellular carcinoma and cholangiocarcinoma in dogs has also shown good outcomes [8, 36]. However, recurrence of cholangiocarcinoma after surgical resection has been reported [50]. Therefore, there is a need for broader knowledge on the mechanism and management of the disease and future therapeutic options for clinicians [51–53].

Because cholangiocarcinoma is reported to respond poorly to chemotherapy, and radiation therapy may be required after surgical approach [26], a new treatment option for advanced biliary tract cancer has been proposed, which is the use of nap-paclitaxel in addition to the gemcitabine and cisplatin combination that is known to be in use [52]. Radiation therapy has become an important component of adjuvant therapy for resected cases and definitive therapy for locally advanced disease [54]. It has been reported that the emerging sophisticated techniques of imaging tumors and conformal dose delivery are increasing the reliability of radiotherapy in the management of bile duct tumors [54]. However, survival is poor due to widespread metastasis to other organs; therefore, there is a need to develop targeted theories and multimodel strategies to improve overall prognosis [55]. The present cases were an advanced form of cholangiocarcinoma, with metastasis to multiple organs and lymph nodes; therefore, radiotherapy was not applied. The dogs may have died from anemia due to hemorrhages, peritonitis and hydroperitoneum, and liver failure, as observed in the hematological and biochemical findings.

## Conclusion

The diagnosis of cholangiocarcinoma in dogs was confirmed through consistent results from clinical signs, cytology, hematology, biochemistry, postmortem examination, and histopathology. The morphological appearance of the tumor showed the mucinous type instead of the most common adenomatous type, and they were diagnosed with fluid samples from lymph nodes in addition to the routine abdominal fluid. Cholangiocarcinoma is found among the canine population in the study location. In dogs exhibiting emaciation, lethargy, icteric mucous membranes, and distended abdomens, cholangiocarcinoma, with a poor prognosis, should be suspected. Appropriate testing, including cytological evaluation of abdominal fluid and lymph node aspirates, hematological analysis, and biochemical analysis, is necessary even without advanced equipment.

## Authors’ Contributions

MFM: Conceptualized the study. MFM, AOL, OMO, and AAA: Designed the study. MAF, KAKF, OEF, OTM, and OMI: Participated in sample and data collection. RSA and AAA: Performed hematological and biochemical analysis. AOL, ASAV, and OMI: Performed the data analysis. MFM: Prepared the first draft of the manuscript. All authors have read, reviewed, and approved the final manuscript.

## References

[1] Nakanuma Y, Kida T, Minato H, Terada T, Tobe T, Kameda H, Okudaira M, Ohto M (1994). Pathology of cholangiocellular carcinoma. Primary Liver Cancer in Japan. Springer-Verlag, Tokyo, Japan.

[2] Al-Bahrani R, Abuetabh Y, Zeitouni N, Sergi C (2013). Cholangiocarcinoma:Risk factors, environmental influences and oncogenesis. Ann. Clin. Lab. Sci.

[3] Selmic L.E (2017). Hepatobiliary neoplasia. Vet. Clin. North Am. Small Anim. Pract.

[4] Nakanuma Y, Uesaka K, Kakuda Y, Sugino T, Kubota K, Furukawa T, Fukumura Y, Isayama H, Terada T (2020). Intraductal papillary neoplasm of bile duct:Updated clinicopathological characteristics and molecular and genetic alterations. J. Clin. Med.

[5] Cullen J.M, Poop J.A, Meuten D.J (2002). Tumours of the liver and gall bladder. Tumours in Domestic Animals.

[6] Clements O, Eliahoo J, Kim J.U, Taylor-Robinson S.D, Khan S.A (2020). Risk factors for intrahepatic and extrahepatic cholangiocarcinoma:A systematic review and meta-analysis. J. Hepatol.

[7] Soyer P, Bluemke D.A, Reichle R, Calhoun P.S, Bliss D.F, Scherrer A, Fishman E.K (1995). Imaging of intrahepatic cholangiocarcinoma:1. Peripheral cholangiocarcinoma. Am. J. Roentgenol.

[8] Kim T.K, Choi B.I, Han J.K, Jang H.J, Cho S.G, Han M.C (1997). Peripheral cholangiocarcinoma of the liver:Two-phase spiral computed tomography findings. Radiology.

[9] Han J.K, Choi B.I, Kim A.Y, An S.K, Lee J.W, Kim T.K, Kim S.W (2002). Cholangiocarcinoma:Pictorial essay of computed tomography and cholangiographic findings. Radiographics.

[10] Doherty B, Nambudiri V.E, Palmer W.C (2017). Update on the diagnosis and treatment of cholangiocarcinoma. Curr. Gastroenterol. Rep.

[11] Lee A.J, Chun Y.S (2018). Intrahepatic cholangiocarcinoma:The AJCC/UICC 8^th^ edition updates. Chin. Clin. Oncol.

[12] Terai K, Ishigaki K, Kagawa Y, Okada K, Yoshida O, Sakurai N, Heishima T, Asano K (2022). Clinical, diagnostic, and pathologic features and surgical outcomes of combined hepatocellular-cholangiocarcinoma in dogs:14 cases (2009–2021). J. Am. Vet. Med. Assoc.

[13] Vogel A, Bridgewater J, Edeline J, Kelley R.K, Klumpen H.J, Malka D, Primrose J.N, Rimassa L, Stenzinger A, Valle J.W, Ducreux M (2023). Biliary tract cancer:ESMO Clinical practice guideline for diagnosis, treatment and follow-up. Ann. Oncol.

[14] Lee S.J, Lim H.K, Jang K.M, Kim S.H, Lee S.J, Lim J.H, Choo IW (2001). Radiologic spectrum of cholangiocarcinoma:Emphasis on unusual manifestations and differential diagnoses. Radiographics.

[15] PetMD (2012). Bile Duct Cancer in Dogs.

[16] Maeda A, Goto S, Iwasaki R, Yamada K, Murakami M, Sakai H, Mori T (2022). Outcome of localized bile duct carcinoma in six dogs treated with liver lobectomy. J. Am. Anim. Hosp. Assoc.

[17] Tanaka T, Noguchi S, Wada Y, Nishida H, Akiyoshi H (2022). Computed tomography findings in canine cholangiocellular carcinoma. Vet. Rec. Case Rep.

[18] Shin D.W, Moon S.H, Kim J.H (2023). Diagnosis of cholangiocarcinoma. Diagnostics (*Basel*).

[19] Stockhaus C, Van den Ingh T, Rothuiizen J, Teske E.A (2004). Multistep approach in the cytologic evaluation of liver biopsy samples of dogs with hepatic diseases. Vet. Pathol.

[20] National Comprehensive Cancer Network (NCCN) (2023). The NCCN Clinical Practice Guidelines in Oncology.

[21] Ersan V, Usta S, Aydin C, Carr B.I, Karatoprak S, Yilmaz S (2023). Critical overview of resection for Bismuth-Corlette type IV perihilar cholangiocarcinoma. Acta Chir. Belg.

[22] Shiga A, Shirota K, Enomoto M (2001). Combined hepatocellular and cholangiocellular carcinoma in a dog. J. Vet. Med. Sci.

[23] Lin C.W, Wu T.C, Lin H.Y, Hung C.M, Hsieh P.M, Yeh J.H, Hsiao P, Huang Y.L, Li Y.C, Wang Y.C, Shu C.W, Chen Y.S (2021). Clinical features and outcomes of combined hepatocellular carcinoma and cholangiocarcinoma versus hepatocellular carcinoma versus cholangiocarcinoma after surgical resection:A propensity score matching analysis. BMC Gastroenterol.

[24] Inchingolo R, Acquafredda F, Ferraro V, Laera L, Surico G, Surgo A, Fiorentino A, Marini S, de'Angelis N, Memeo R, Spiliopoulos S (2021). Non-surgical treatment of hilar cholangiocarcinoma. World J. Gastrointest. Oncol.

[25] Ruff S.M, Cloyd J.M, Pawlik T.M (2023). Annals of surgical oncology practice guidelines series:Management of primary liver and biliary tract cancers. Ann. Surg. Oncol.

[26] Chamberlain R.S, Blumgart L.H (2000). Hilar cholangiocarcinoma:A review and commentary. Ann. Surg. Oncol.

[27] Spee B, Jonkers M.D, Arends B, Rutteman G.R, Rothuizen J, Penning L.C (2006). Specific downregulation of XIAP with RNA interference enhances the sensitivity of canine tumor cell-lines to TRAIL and doxorubicin. Mol. Cancer.

[28] Yu H.B, Ge C.L, Huang Z.H, Wang H, Liu Z.Y, Zhang J.R (2009). Effect of targeted argon-helium cryoablation on the portal region in canine livers. Nan Fang Yi Ke Da Xue Xue Bao.

[29] Balkman C (2009). Hepatobiliary neoplasia in dogs and cats. Vet. Clin. North Am. Small Anim. Pract.

[30] Colville J, Hendrix C.M (2002). Blood chemistry. Laboratory Procedures for Veterinary Technicians.

[31] Doumas B.T, Kwok-Cheung P.P, Perry B.W, Jendrzejczak B, McComb R.B, Schaffer R, Hause L.L (1985). Candidate reference method for determination of total bilirubin in serum:development and validation. Clin. Chem.

[32] Blechacz B.R.A, Gores G.J (2008). Cholangiocarcinoma. Clin. Liver Dis.

[33] Post G, Patnaik A.K (1992). Nonhematopoietic hepatic neoplasms in cats:21 cases (1983–1988). J. Am. Vet. Med. Assoc.

[34] Sammadder N.J, Smith K.R, Wong J, Hansan H, Bouchr K, Burt R.W, Charlton M, Byrne K.R, Gallegos-Orozco J.F, Koptiuch C, Curtin K (2016). Familial risk of biliary tract cancers:A population-based study in Utah. Dig. Dis. Sci.

[35] Thamm D.H, Withrow S.J, MacEwen E.G (2001). Hepatobiliary tumours. Small Animal Clinical Oncology.

[36] Choi B.I, Han J.K, Hong S.T, Lee K.H (2004). Clonorchiasis and Cholangiocarcinoma:Etiologic relationship and imaging diagnosis. Clin. Microbiol. Rev.

[37] Hammer A.S, Sikkema D.A (1995). Hepatic neoplasia in the dog and cat. Vet. Clin. North Am. Small Anim. Pract.

[38] Yamamoto S, Kubo S, Hai S, Uenishi T, Yamamoto T, Shuto T, Takemura S, Tanaka H, Yamazaki O, Hirohashi K, Tanaka T (2004). Hepatitis C virus infection as a likely etiology of intrahepatic cholangiocarcinoma. Cancer Sci.

[39] Munro R, Young R.W (1996). Hepatocellular tumours in roe deer in Britain. Vet. Rec.

[40] Moore A.S, Ogilvie G.K (2001). Splenic, hepatic and pancreatic tumours. Feline Oncology. Veterinary Learning Systems, W.B. Saunders, Trenton, NJ, Philadelphia, PA.

[41] Liptak J.M, Dernell W.S, Withrow A.S (2004). Liver tumours in cats and dogs. Compend. Contin. Educ. Pract. Vet.

[42] Ka I, Faye M, Diop P.S, Faye A.B.N.A.C, Ndoye J.M, Fall B (2018). Clinical, epidemiological and therapeutic features of biliary tract cancers:About 20 cases. Pan Afr. Med. J.

[43] Aslan O, Culcir L, Berdik I.K, Dogan Z, Tunc A.S (2014). Cholangiocarcinoma of intrahepatic bile ducts with disseminated metastases in a Siamese cat:A case report. Vet. Med.

[44] Gong Z.J, Cheng J.W, Gao P.T, Huang A, Sun Y.F, Zhou K.Q, Hu B, Qiu S.J, Zhou J, Fan J, Yang X.R (2019). Clinical characteristics and prognostic factors of patients with intrahepatic cholangiocarcinoma with fever:A propensity score matching analysis. Oncologist.

[45] Pereira-Lima J.C, Jakobs R, Busnello J.V, Benz C, Blaya C, Riemann J.F (2000). The role of serum liver enzymes in the diagnosis of choledocholithiasis. Hepatogastroenterology.

[46] Bain V.G, Abraham N, Jhangri G.S, Alexander T.W, Henning R.C, Hoskinson M.E, Maguire C.G, Lalor E.A, Sadowski D.C (2000). Prospective study of biliary structures to determine the predictors of malignancy. Can. J. Gastroenterol.

[47] Alvaro D, Benedetti A, Marucci L, Delle Monache M, Monterubbianesi R, Di Cosimo E, Perego L, Macarri G, Glaser S, Le Sage G, Alpini G (2000). The function of alkaline phosphatase in the liver:Regulation of intrahepatic biliary epithelium secretory activities in the rat. Hepatology.

[48] Ciftci M.K, Ortatatli M, Avki S (1998). Cholangiocellular carcinoma in a cat (in Turkey). Vet. Bilimler. Derg.

[49] Weber A, Schmid R.M, Prinz C (2008). Diagnostic approaches for cholangiocarcinoma. World J. Gastroenterol.

[50] Zhang X.F, Beal E.W, Chakedis J, Chen Q, Lv Y, Ethun C.G, Salem A, Weber S.M, Tran T, Poultsides G, Son A.Y, Hatzaras I, Jin L, Fields R.C, Buettner S, Scoggins C, Martin R.C.G, Isom C.A, Idrees K, Mogal H.D, Shen P, Maithel S.K, Schmidt C.R, Pawlik T.M (2018). Defining early recurrence of hilar cholangiocarcinoma after curative-intent surgery:A multi-institutional study from the US extrahepatic biliary malignancy consortium. World J. Surg.

[51] Banales J.M, Marin J.J.G, Lamarca A, Rodrigues P.M, Khan S.A, Roberts L.R, Cardinale V, Carpino G, Andersen J.B, Braconi C, Calvisi D.F, Perugorria M.J, Fabris L, Boulter L, Macias R.I.R, Gaudio E, Alvaro D, Gradilone S.A, Strazzabosco M, Marzioni M, Coulouarn C, Fouassier L, Raggi C, Invernizzi P, Mertens J.C, Moncsek A, Ilyas S.I, Heimbach J, Koerkamp B.G, Bruix J, Forner A, Bridgewater J, Valle J.W, Gores G.J (2020). Cholangiocarcinoma 2020:The next horizon in mechanisms and management. Nat. Rev. Gastroenterol. Hepatol.

[52] Mizrahi J.D, Shroff R.T (2020). New treatment options for advanced biliary tract cancer. Curr. Treat. Options Oncol.

[53] Moris D, Palta M, Kim C, Allen P.J, Morse M.A, Lidsky M.E (2023). Advances in the treatment of intrahepatic cholangiocarcinoma:An overview of the current and future therapeutic landscape for clinicians. CA Cancer J. Clin.

[54] Bridgewater J.A, Goodman K.A, Kalyan A, Mulcahy M.F (2016). Biliary tract cancer:Epidemiology, radiotherapy, and molecular profiling. Am. Soc. Clin. Oncol. Educ. Book.

[55] Padmanaban V, Ruff S.M, Pawlik T.M (2024). Multidisciplinary care of hilar cholangiocarcinoma:Review of guidelines and recent advancements. Cancers (*Basel*).

